# Analysis of Allergy and Hypersensitivity Reactions to COVID-19 Vaccines According to the EudraVigilance Database

**DOI:** 10.3390/life14060715

**Published:** 2024-05-31

**Authors:** Jan Romantowski, Wojciech Nazar, Kinga Bojahr, Iwona Popiołek, Marek Niedoszytko

**Affiliations:** 1 Department of Allergology, Medical University of Gdansk, 80-210 Gdansk, Polandmnied@gumed.edu.pl (M.N.); 2 Faculty of Medicine, Medical University of Gdańsk, 80-210 Gdańsk, Poland; wojciech.nazar@gumed.edu.pl; 3 Department of Toxicology and Environmental Diseases, Jagiellonian University Medical College, 31-008 Krakow, Poland; iwona.popiolek@uj.edu.pl

**Keywords:** vaccination, drug allergy, anaphylaxis, rash, side effect, adverse drug reaction, COVID-19, European Economic Area

## Abstract

Background: The coronavirus disease 2019 (COVID-19) pandemic presented a new challenge in modern medicine: the development of vaccines was followed by massive population vaccinations. A few reports on post-vaccination allergic reactions have made patients and medical personnel uneasy as to COVID-19 vaccines’ allergic potential. Most of the studies in this area to date have been small, and some that were based on global databases skipped most of the allergic diseases and concentrated only on anaphylaxis. We aimed to analyze the incidence of serious allergic reactions based on the EudraVigilance (EV) database, regardless of the reported symptoms and allergy mechanism. Methods: The total number of administrated vaccine doses was extracted on 5 October 2023 from Vaccine Tracker and included all administrations since vaccinations began in the European Economic Area (EEA). Data on serious allergic reactions to COVID-19 vaccines were extracted from the EudraVigilance database with the same time point. The code names of 147 allergic symptoms or diseases were used. Results: The frequency of serious allergic reactions per 100,000 administered vaccine doses was 1.53 for Comirnaty, 2.16 for Spikevax, 88.6 for Vaxzevria, 2.11 for Janssen, 7.9 for Novavax, 13.3 for VidPrevtyn Beta, and 3.1 for Valneva. The most prevalent reported reactions were edema (0.46) and anaphylaxis (0.40). Only 6% of these reactions were delayed hypersensitivity-oriented. Conclusions: The overall frequency of potential serious allergic reactions to COVID-19 is very rare. Therefore, COVID-19 vaccines seem to be safe for human use. The lowest frequency of allergic reaction was observed for Comirnaty and the highest for Vaxzevria.

## 1. Introduction

The coronavirus disease 2019 (COVID-19) pandemic that started in 2019 generated new challenges in modern medicine [1]. Initially, no effective treatment was available and the medical focus was on disease prevention: (1) passive, with social distancing and personal protection, and (2) active, with vaccinations [2]. The ensuing rapid, one-year-long vaccine development process has made societies uneasy about vaccination safety [3,4,5,6]. Although product characteristics and medical society guidelines, such as the European Academy of Allergy and Clinical Immunology (EAACI), highlighted the robust safety and low allergenicity of anti-COVID-19 vaccines, reports on anaphylactic reactions still resulted in vaccination reluctance, especially in patients who had experienced drug-related hypersensitivities in the past [1,7,8]. The available COVID-19 vaccines may cause cutaneous adverse effects, including injection site reactions, urticaria, angioedema, the exacerbation of atopic eczema, and even anaphylaxis [9]. The severe hypersensitivity symptoms mostly started within 10–30 min of exposure. The most common symptoms were urticaria, itching, flushing, angioedema, shortness of breath, a burning sensation, and fainting [10]. As the population vaccination process continued, more data were available and hypersensitivity reactions were estimated at 0.66% of doses, with the majority of these being immediate reactions (0.53%) [11]. Delayed hypersensitivity was observed in 0.1% of doses. With the preventive implementation of skin prick tests and intradermal tests for vaccines and excipients, it was possible to vaccinate over 99% of patients with a high risk of allergic reactions [12,13,14]. Even in cases of severe delayed allergic reactions, new in vitro tests were developed to enable vaccination, with promising results for the future [15].

EudraVigilance (EV) is a European database supervised by the European Medicines Agency (EMA) that gathers reported suspected side effects of drugs that are authorized for users in the European Economic Area [16]. It is open-access for all users. A single adverse event report can be submitted by anyone, though they are categorized by reporter, as (1) healthcare professional and (2) non-healthcare professional. Apart from the name of the adverse reaction, they are also categorized by seriousness, as (1) serious and (2) non-serious. By definition, adverse events include all harmful symptoms that may have been caused by drugs, even including overdoses. This freedom of reporting can also be perceived as a flaw that results in the appearance of symptoms that are almost impossible to logically connect to the administered drug. It is also worth noting that EV does not gather reports itself. All adverse events are reported to national medical agencies and then transferred digitally to EV. This may result in discrepancies in methodologies, which may affect the final outcome. On the other hand, the database contains such a great number of cases that those flaws might become irrelevant.

COVID-19 Vaccine Tracker is an open-access tool operated by the European Centre of Disease Control and Prevention [17]. It gathers information on the number of anti-COVID-19 vaccines administrated in the European Economic Area. The doses are categorized by the name of the vaccine and country. The report ends on 5 October 2023 with a total of 981,454,243 doses administered. The number of administered doses can be used for adverse event incidence calculations.

To date, there have been a few reports on the incidence of hypersensitivity and allergic reactions after COVID-19 vaccines. Most of them concentrated on specific populations, such as children, asthmatics, or patients with mastocytosis, and thoroughly described allergic reactions. These were rather limited to a few thousand patients [18,19,20,21,22,23]. A few reports on large databases such as EV were published, though these concentrated mainly on anaphylaxis or anaphylactic shock as life-threatening reactions, which are easily coded in the database [24,25,26,27,28,29,30,31]. Other reactions, such as edema, urticaria, and maculopapular rash, are difficult to code and thus to find all instances of in the database. In EV, they might also appear as ‘swelling’, ‘blistering’, ‘rash’, or ‘allergy’, especially when they are reported by someone who is not a healthcare professional. Popiołek et al. successfully created a list of reaction names that correspond to the most likely allergic reactions [32]. The list includes 147 names of reactions that are available for filtering in the EV database. They are presented in the Supplementary Materials and also categorized according to the affected organs and suspected mechanism (for example, type I or IV hypersensitivity according to the EAACI classification) [33]. The study aimed to identify, analyze, and evaluate the frequency of hypersensitivity reactions to anti-COVID-19 vaccines available in the European Economic Area based on the EudraVigilance database and vaccination tracker.

## 2. Materials and Methods

The total number of administrated vaccine doses was extracted on 5 October 2023 from Vaccine Tracker [17]. The data for all COVID-19 vaccines shown in Table 1 were extracted on 6 December from the EudraVigilance database using a line listing export for all serious adverse events [16]. Serious adverse events include those that are life-threatening, result in death, require hospitalization, prolong hospitalization, or result in disability or congenital defect.

During the analyzed period, a total of 567,203,616 doses of Comirnaty, 132,734,949 doses of Spikevax, 56,007,792 doses of Vaxzevria, 16,056,640 doses of Janssen, 225,312 doses of Novavax, 7524 doses of VidPrevtyn Beta, and 2257 doses of Valneva were distributed in the EEA region.

### Statistical Analysis

To calculate the frequency of each allergic reaction for the given vaccine, the reported number of cases for every allergic reaction was divided by the total number of doses administered in EU/EEA countries. Moreover, selected reactions that had a similar description of the represented symptom/pathology were grouped into main categories. A detailed table showing the grouping of reactions into main categories is available in the Supplementary Materials.

Categorical data were summarized with the use of frequencies. The chi-square test with Yates’s correction was used to check for the statistical significance of the differences between the grouping variables. The threshold of statistical significance was set at *p* < 0.05. The analyses were performed in Python 3.10 using the Numpy, Pandas, and Scipy libraries.

## 3. Results

Initially, there were 945,909 records in the EudraVigilance database on 6 December related to this study. All records added after 5 October 2023 were removed (*n* = 15,489). Next, all records from non-European economic areas were also removed (*n* = 505,958). Thus, the records in the EUDRA database and the COVID-19 Vaccine Tracker database of the European Centre for Disease Control and Prevention referred to the same period and were collected from the same region (European Economic Area). Next, all records that were not considered as an ‘allergic reaction’ were also removed from the database (*n* = 410,793). There were no missing data. In total, there were 13,669 records remaining. These records were further analyzed statistically.

### 3.1. General Characteristics

**Figure 1:**
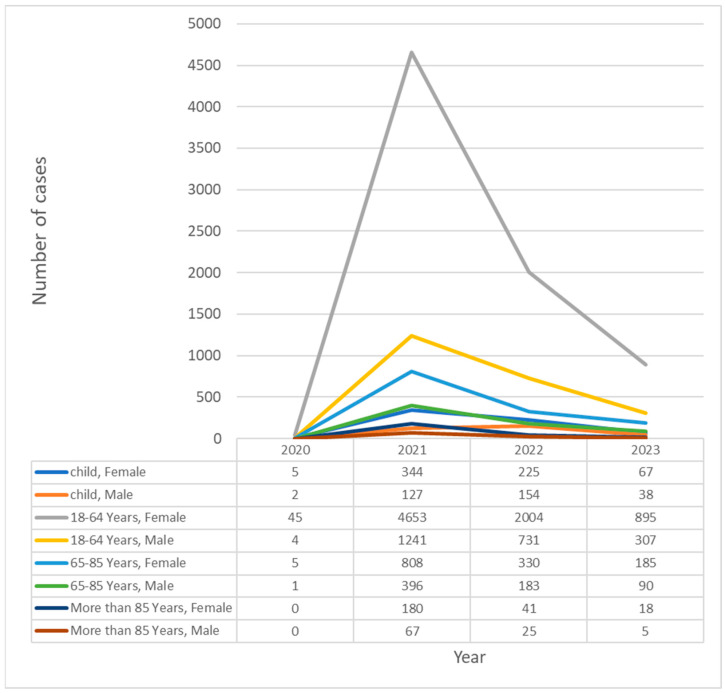
Number of cases of all serious allergic reactions post COVID-19 vaccination according to year of occurrence in each age and sex group. Child—person under 18 years old.

The dataset included 113 types of allergic reactions (*n* = 13,669). Adverse reactions to the Comirnaty (*n* = 8734), Spikevax (*n* = 2857), Vaxzervria (*n* = 1717), Janssen (*n* = 340), Novavax (*n* = 18), VidPrevtyn Beta (*n* = 1), and Valneva (*n* = 2) vaccine products were analyzed. When divided by the total number of doses administered in the EEA region for each vaccine, the frequencies of allergic reactions per 100,000 doses to the analyzed vaccines were 1.53 for Comirnaty, 2.16 for Spikevax, 3.1 for Vaxzevria, 2.16 for Janssen, 7.99 for Novavax, 88.6 for Valneva, and 13.3 for VidPrevtyn Beta. It must be noted that Novavax, VidPrevtyn Beta, and Valneva had far fewer administrations (below 1,000,000); thus, the incidence calculations might be uncertain. The results in subgroups according to sex and age are presented in Table 2. In these subgroups, the most reactions occurred in women aged 18–64. In Figure 1, the cases are divided according to the year of report, which shows that despite the greatest number of cases being reported in 2021 during the massive, whole-population vaccination, significant numbers were still reported in 2023, probably mostly in people receiving booster doses. In statistical analysis using chi-square contingency tables, all comparisons in vaccine, sex, and age groups were statistically significant (*p* < 0.001) due to large sample sizes.

### 3.2. Type of Reaction

**Figure 2:**
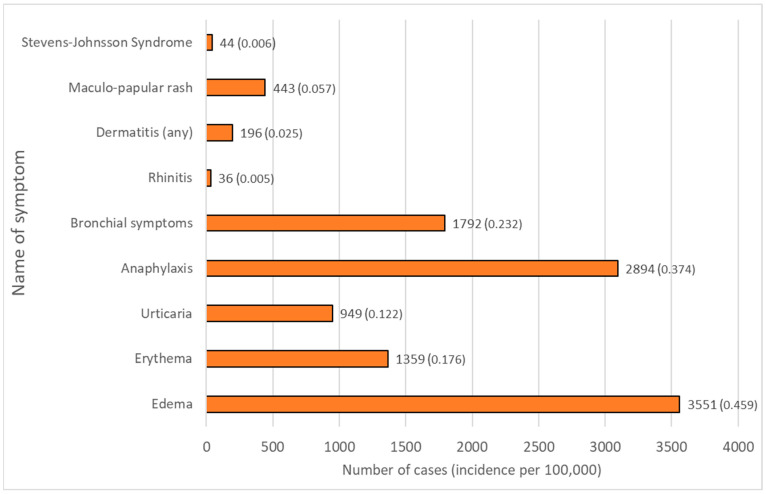
Total number of main allergic reactions categories to COVID-19 vaccinations. Similarly described reactions have been grouped together for better readability (for grouping information, see Supplementary Materials Table S1). Incidences per 100,000 are provided in brackets.

All reactions were divided according to the time of onset, which also corresponds to the suspected mechanism: (1) immediate reactions (*n* = 10,853), (2) delayed reactions (*n* = 830), (3) unknown time reactions (*n* = 1986). Unknown time reactions included ‘erythema’, ‘hypersensitivity’, ‘rash’, ‘drug eruption’, ‘injection site reaction’, ‘drug hypersensitivity’, and ‘allergic reaction to excipient’. These code names of reactions were impossible to classify into either immediate or delayed hypersensitivity and, due to the construction of the EV database, no additional information is available. The most clinically important reactions according to the number of cases are presented in Figure 2.

### 3.3. Differences in Allergy and Hypersensitivity Reactions between Vaccines

When all vaccinations against COVID-19 are considered, the most frequently observed main categories of postvaccination allergic and hypersensitivity reactions were edema (*n* = 3551), followed by anaphylaxis (*n* = 2894), bronchial symptoms (*n* = 1792), urticaria (*n* = 949) and rhinitis (*n* = 36). Moreover, the ten most commonly observed specific types of reactions after the vaccine administration were an anaphylactic reaction (*n* = 2018), angioedema (*n* = 1564), cough (*n* = 1459), erythema (*n* = 1359), urticaria (*n* = 949), hypersensitivity (*n* = 893), anaphylactic shock (*n* = 787), rash (*n* = 769), blister (*n* = 276), and erythema multiforme (*n* = 261, Table 3). For all of them, statistically significant differences in the incidence rates between studied vaccines were observed (*p* < 0.05).

When it comes to the most prevalent reactions after the administration of each studied vaccine, for Comirnaty, the most frequently administered vaccine, anaphylactic reaction, angioedema, and cough were the most commonly observed reactions and had an incidence of 0.258, 0.183, and 0.172 per 100,000 vaccine doses administered (VDA), respectively (Table 3). Vaccination with Spikevax was most frequently associated with the risk of erythema, urticaria, and angioedema (0.405, 0.249, and 0.216 per 100,000 VDA, respectively). After the administration of Vaxzevria anaphylactic reaction, erythema, and angioedema were the most prevalent reactions (0.454, 0.368, and 0.364 per 100,000 VDA). Anaphylactic reaction, cough, and anaphylactic shock were the most common complications of vaccination with Janssen (0.374, 0.287, and 0.287 per 100,000 VDA, respectively). The administration of Novavax had the highest risk of an anaphylactic reaction, hypersensitivity, and rash (1.775, 1.332, and 1.332, per 100,000 VDA, respectively).

The administration of Valneva was followed by urticaria or anaphylactic reaction, each observed with an incidence rate of 44.307 per 100,000 VDA. After vaccination with VidPrevtyn, erythema was observed (13.291 per 100,000 VDA).

## 4. Discussion

The results of our study highlight the safety of COVID-19 vaccines. Serious hypersensitivity events occur in 1.5 cases per 100,000 doses for Comirnaty, up to 88 per 100,000 for Vaxzevria. Our data also highlight possibly the most at-risk population as being adult women < 65 years old. The low number of allergic cases reported in the elderly is fortunate, while this group is considered the most vulnerable to COVID-19 [35]. The rationale behind limiting the results to serious events was that the EAACI considers only serious allergic reactions as a potential contraindication for further vaccinations [1]. In addition, non-serious adverse events would include vast numbers of insignificant local reactions and typical side effects, such as increased body temperature [36].

The total rates of allergic reactions per 100,000 inoculations for the analyzed vaccines were as follows: 1.53 for Comirnaty, 2.16 for Spikevax, 3.07 for Vaxzevria, 2.16 for Janssen, 7.99 for Novavax, 88.6 for Valneva, and 13.3 for VidPrevtyn Beta. It should be noted that Novavax, VidPrevtyn Beta, and Valneva had significantly fewer administrations (less than 1,000,000), which may make the incidence calculations unreliable and the incidence rates might be overestimated (Table 2).

EMA groups the frequency of adverse drug reactions to the authorized medicines and vaccines as follows: very common (≥1/10); common (≥1/100 to <1/10); uncommon (≥1/1000 to <1/100); rare (≥1/10,000 to <1/1000 or ≥10/100,000 to <100/100,000); very rare (<1/10,000 or <10/100,000); and “frequency not known (cannot be estimated) from the available data” [37].

According to our study, the total incidence rates per 100,000 VDA were below 10 for Comirnaty, Spikevax, Vaxzevria, Janssen, and Novavax, which classifies the frequencies of allergic reactions to these vaccines as “very rare”. For Valneva and VidPrevtyn Beta, for which the total incidence rates per 100,000 VDA were over 10 and below 100, and the total frequency of allergic reactions should be classified as “rare”. Thus, based on this data, the use of any COVID-19 vaccine seems to be safe. The mRNA vaccines (Comirnaty and Spikevax) seem to have a better overall safety profile than vector-based and protein-based vaccines (Vaxzevria, Janssen, Novavax, Valneva, and VidPrevtyn Beta). Among the non-mRNA-based vaccines, Janssen and Vaxzevria have the best allergic safety profile. The Janssen vaccine’s safety performance even matches that of Spikevax, as both vaccines have a total frequency of allergic events equal to 2.16 per 100,000 VDA [38].

Considering all COVID-19 vaccinations, the ten most frequently reported allergic and hypersensitivity reactions were anaphylactic reaction, angioedema, cough, erythema, urticaria, hypersensitivity, anaphylactic shock, rash, blister, and erythema multiforme (Table 3). These adverse drug reactions represent the most common clinical manifestations of acute allergic reactions and anaphylactic shock in general, as described by the World Allergy Organization [39].

However, when it comes to the specific serious allergic events that were observed for the vaccines analyzed in our study, there are statistically significant differences between the studied substances (Table 3). Therefore, each vaccine has clinically different manifestations of potential allergic reactions that might occur after the vaccination.

The general incidence results are in line with other studies. According to Chu et al.’s meta-analysis of almost 1400 people who had an adverse reaction to the COVID-19 vaccination’s first dosage, just 6 people (0.4%) had severe reactions after receiving their second dose, while 232 people (17%) only had mild symptoms [40]. In December 2020, the initial US surveillance data revealed a hypersensitivity incidence of 11.1 cases per million doses for the Pfizer-BioNTech COVID-19 vaccine and 2.5 cases per million doses for the Moderna vaccine, with lower rates more recently [41,42]. According to data from Vaccine Safety Datalink Overall, the incidence of COVID-19 mRNA vaccine-associated anaphylaxis is very low, with only 4.8 per million doses for BNT162b2 and 5.1 per million doses for mRNA-1273 [43]. The prevalence of anaphylaxis associated with COVID-19 vaccinations currently appears to be comparable to those observed with other vaccinations. The difference in comparison between vaccines might be due to the time of publication. Studies published at the beginning of massive vaccinations included mostly initial doses, while current studies (including ours) also take into account boosters. This might increase the incidence and slightly change the relations between vaccines, as more and more people are being exposed to the regimen and have the opportunity to develop an allergy.

Before widespread COVID-19 immunization campaigns started, serious allergic reactions, including anaphylaxis associated with vaccines, were believed to be extremely unusual events. For instance, between January 2009 and December 2011, the rate of anaphylaxis was 1.31 per million vaccination doses [44].

A commonly known issue is the fact that any adverse reaction experienced in connection with vaccination, such as nausea or subjective oropharyngeal symptoms, which is difficult to evaluate during a physical examination is frequently labelled as an allergy [45]. Hourihane et al. reassessed documented cases using the Brighton Collaboration Criteria and criteria by the National Institute of Allergy and Infectious Diseases (NIAID) 2005. They found that 71% of cases previously reported as anaphylaxis were reclassified as not satisfying anaphylaxis criteria [46]. This would mean that the incidence of allergic reactions in datasets that include self-reporting would be overestimated. However, due to the large dataset, comparing between particular vaccines is still viable.

The study by Kyeonghun and colleagues was a large-scale survey based on the WHO database, covering approximately 55 years. They reported that vaccination for DTaP-IPV-Hib is the most common cause of vaccine-associated anaphylaxis and occurs much more frequently [47]. Importantly, despite the extreme rarity of reaction, they found that vaccines presented a relatively high fatality rate once anaphylaxis occurred. The highest fatality rate (15.0%) was associated with the Ad5-vectored COVID-19 vaccine, followed by tuberculosis (9.6%), COVID-19 mRNA (7.2%), cholera (6.3%), influenza (5.8%), encephalitis (3.3%), and hepatitis B (2.4%). In contrast, according to the Vaccine Adverse Event Reporting System in the USA, only 8 (0.97%) of 828 cases of vaccine-related anaphylaxis resulted in death [48]. Such a discrepancy is difficult to explain and might be a result of the great variety of reporting processes around the world. The most important conclusion from this vast analysis is that, among other vaccines, anti-COVID-19 regimens present even fewer hypersensitivity reactions.

Still, it is important to analyze the proportions of each type of reaction, as most of them (73%) are of the immediate type and respond relatively well to standard treatments [49]. Only 6% (see Figure 1) of all serious reactions were delayed, such as SJS, TEN, or maculopapular rash, with difficult treatment and diagnostic processes [15]. These may be patients who cannot undergo standard allergy work-up protocols and thus may be disqualified from further vaccinations.

A higher incidence of hypersensitivity to vaccines and other drugs in women has been described before [12,50,51,52,53]. The reason for this phenomenon remains unclear. Possible explanations include the epigenetics of the X chromosome, higher use of medications in women, longer elimination time, and the role of sex hormones. It is also possible that reporting bias contributes to this discrepancy to some extent. However, Watson et al. claim that though women report more drug-related adverse events in general, serious and fatal adverse events are more likely to be reported in men [54].

It is worth comparing the incidence of vaccine allergy with other commonly used drugs. Beta-lactam antibiotics (BL), including penicillins, cephalosporins, monobactams, and carbapenems, are the most widely used antimicrobials because of their effectiveness and safety. Nevertheless, they are also the most prevalent cause of drug-induced hypersensitive reactions [55,56,57]. The penicillin allergy remains the most frequently reported drug allergy. It is estimated that 5–10% of the general public, and up to 19% of hospitalized patients, have a beta-lactam allergy; however, this is confirmed in only 10% of patients [58]. The frequency of beta-lactam allergy reports has grown throughout the years, rising from 1–2% in 1980 to 5–13% in the most recent decades [56,59,60,61]. Patients who receive more health care, particularly women and the elderly, have higher rates [53,62]. The incidence of anaphylaxis caused by penicillin is estimated to be between 0.015% and 0.04%, with fatality rates ranging between 0.0015% and 0.002% [63,64]. It is estimated that 3% of cases of drug-induced anaphylaxis are fatal [65].

Another common cause of antibiotic allergy is sulfonamide antibiotics. Giles et al. pointed out that sulfonamide allergies are reported in approximately 3–8% of patients in the general population [66]. A sulfonamide allergy can present with a wide range of clinical symptoms. These can include delayed cutaneous maculopapular eruptions, which is the most common manifestation of sulfonamide allergy [66,67]. The most prominent known risk factor for a sulfonamide allergy is being persistently HIV positive, especially in those with AIDS [67]. According to Carr et al., 27% of HIV patients treated with trimethoprim/sulfamethoxazole (TMP-SMX) for pneumocystis pneumonia experienced hypersensitivity responses [68].

Iodinated contrast media (ICM) generates both immediate and delayed hypersensitivity reactions [69,70]. In a multicenter study of 196,081 patients in Korea, the prevalence of HSR in those who underwent ICM administration was 0.73%, while severe reactions occurred in 0.01%. Regarding severity, 83.2% of the occurrences were categorized as mild HSRs, with a total incidence of 0.61% (1192 of 196,081), 15.6% as moderate HSRs (0.11%; 224 of 196,081), and 1.2% as severe HSRs [71]. This study showed a comparable, or lower, prevalence of HSR compared to other large studies [72,73].

A large study conducted by Voltolini and colleagues investigated the medical records of patients receiving care in nine Italian allergy centers for experiencing hypersensitivity reactions to ICM and compared them with a control group of 152 subjects that tolerated one or more ICM-enhanced examinations. As described in previous studies, females and patients under 65 years of age were more likely to experience hypersensitive reactions. Furthermore, potential risk factors for ICM reactions include respiratory allergies and cardiovascular illness, and include a previous reaction, chronic disorders, a history of asthma, adverse drug reactions and food allergy, and a family history of radiologic contrast media reaction [74].

Nonsteroidal anti-inflammatory medications (NSAIDs) belong to the most commonly used medications worldwide [75], which may explain why hypersensitivity reactions to NSAIDs are quite prevalent. NSAID-induced hypersensitivity can range in severity from mild symptoms to severe, potentially life-threatening anaphylaxis [76]. The prevalence of hypersensitivity reactions to NSAIDs in the general population ranges from 0.2% up to 0.6% [77]. However, there are certain patient groups that present much higher hypersensitivity: patients with asthma (21%) or chronic urticaria (20–40%) [78,79,80].

Zhou et al. analyzed drug allergy data obtained from the electronic health records of 1,766,328 patients who visited hospitals in Boston from 1990 to 2013. They found that NSAIDs ranked as the fourth most frequently reported drug allergy, with aspirin being the most commonly reported in this group [59,81].

Aspirin has been reported to be the most prevalent NSAID allergy in certain studies, whereas naproxen has been found to be the most common allergy in others [81,82].

In a 6-year study involving almost 4500 patients by Doña et al., NSAIDs were the drugs most frequently linked to HSRs, with ibuprofen being the most frequent [83].

NSAIDs were the most often reported analgesic allergy until 1993. Opioids, however, are now the allergy that is reported most often. Up to 2006, NSAIDs accounted for just 5–8% of reported drug allergies, while opioids accounted for 10–15% [59].

Local anesthetics (LAs) are widely used drugs, especially in delivery, dentistry, and surgery. During anesthesia and surgery, allergic reactions to LAs, particularly anaphylaxis, can be life-threatening. However, numerous studies support the extremely low incidence of IgE-mediated allergic reactions, which are thought to be responsible for less than 1% of all reported reactions [58,84,85]. Kvisselgaard et al. claim that various nonallergic mechanisms are usually the main cause of adverse drug reactions to Las [86]. The vasovagal response, which can cause syncope and hypotension, overdose toxicity with paresthesia, and incorrect medication administration are a few of these [87].

During a period of 10 years, Kvisselgaard and colleagues examined the frequency of IgE-mediated immediate-type perioperative allergic reactions to LAs. They found that out of 162 patients who were suspected of having perioperative allergic reactions and had been exposed to an LA, none of them responded to the relevant LA on subcutaneous provocation. As a result, from 2004 to 2013, no patients in the Danish Anaesthesia Allergy Centre have been diagnosed with an LA allergy [88].

Out of 135 events, only two (1.5%) were identified as hypersensitive to an LA in the Harboe et al. study. IgE-mediated allergies to other substances, such as latex, triamcinolone, chlorhexidine, and potentially hexaminolevulinate, were identified in ten reactions (7%) [84].

According to research by Zuo et al., only 6 of 109 patients who were referred to a Chinese anesthesia allergy clinic and had allergy tests performed with LAs during a ten-year period really had a true allergy to LAs. Out of those patients, the culprit drug was lidocaine in four cases, and ropivacaine in three [89].

The comparison with other drug groups presented above favors COVID-19 vaccines, especially in regards to typically used drugs, such as NSAIDs and beta-lactams. Even among other vaccines, COVID-19 vaccines seem safe and the risk/benefit assessment strongly suggests vaccinations in patients with no post-vaccine allergic reactions. The strengths of our study are (1) large numbers covering most of the exposed population and (2) real-life data unaffected by selection bias. The limitation of our analysis is its retrospective method. Thus, we were not able to classify the details of all of the described reactions in terms of etiology and timing.

## 5. Conclusions

COVID-19 vaccines are safe, especially compared to other vaccines and other groups of pharmaceuticals, as presented above. This information could be supplied to the patient hesitating before vaccination, along with the statement that if a person with no allergic history does not hesitate to take antibiotics, vaccination should also not pose an allergy-related issue. The incidence ranges from 1.5 up to 88 per 100,000 administered doses, with Comirnaty being the safest. The highest number of allergic reactions was reported in adult women <65 years old. Even though the risk of having an allergic reaction is low, given the very large number of administered vaccines, medical personnel, particularly allergologists, need to be prepared to properly handle potential allergic reactions.

## Figures and Tables

**Table 1:** Investigated vaccines by substance name as they appear in the EudraVigilance database [34].

Manufacturer Name/Commonly Used Name						
Moderna/ Spikevax	Pfizer-BioNTech/ Comirnaty	AstraZeneca/Vaxzevria	Johnsson/Janssen	Novavax	Valneva	VidPrevtyn
elasomeran	tozinameran	CHADOX1 NCOV-19	AD26.COV2.S	NVX-COV2373	Valneva	VidPrevtyn
andusomeran	raxtozinameran					
imelasomeran	riltozinameran					
davesomeran	famtozinameran					

**Table 2:** Comparison of number of allergic reactions according to vaccine, age, and sex. Due to reporting discrepancies, the general population cases might not be the sum of the subgroups, such as female and male, as it is possible that some patients’ characteristics were not specified in some reports. *p* value was calculated using chi-square. All comparisons in vaccines and age groups were statistically significant due to large sample sizes.

Number of Cases	Spikevax	Comirnaty	Vaxzevria	Janssen	Novavax	Valneva	VidPrevtyn Beta
General population	2857	8734	1717	340	18	2	1
Female	2052	6363	1216	157	15	1	1
Male	753	2064	409	141	3	1	0
Female, child (<18 years old)	89	479	65	6	1	0	1
Male, child (<18 years old)	61	236	14	9	1	0	0
Male, Adult 18–64 years old	572	1322	263	123	2	1	0
Female, Adult 18–64 years old	1647	4870	928	137	14	1	0
Female, elderly >85	27	202	10	0	0	0	0
Male, elderly >85	14	78	3	2	0	0	0
Female Adult 65–85 years old	289	812	213	14	0	0	0
Male Adult 65–85 years old	106	428	129	7	0	0	0
Total incidence per 100,000	Spikevax	Comirnaty	Vaxzevria	Janssen	Novavax	Valneva	VidPrevtyn Beta
General population	2.16	1.53	3.07	2.16	7.99	88.6	13.3

**Table 3:** Incidence per 100,000 administered vaccine doses of allergy and hypersensitivity reactions to vaccines against COVID-19. Statistically significant differences (*p* < 0.05) are bolded. The table includes only reactions with total *n* > 100. The frequencies of all reactions and a table showing the grouping of reactions into main categories are available in the Supplementary Materials.

Main Categories									
Reaction	*n*	Spikevax	Comirnaty	Vaxzevria	Janssen	Novavax	Valneva	VidPrevtyn	*p*-Value
Bronchial symptoms	1792	0.228	0.213	0.407	0.318	0.888	0.000	0.000	<0.001
Anaphylaxis	2894	0.254	0.367	0.645	0.666	2.663	44.307	0.000	**<0.001**
Rhinitis	36	0.003	0.005	0.007	0.013	0.000	0.000	0.000	0.9805
Urticaria	949	0.249	0.090	0.157	0.106	0.000	44.307	0.000	**<0.001**
Edema	3551	0.539	0.403	0.841	0.473	0.888	0.000	0.000	**<0.001**
All reactions									
Anaphylactic reaction	2018	0.176	0.258	0.454	0.374	1.775	44.307	0.000	**<0.001**
Angioedema	1564	0.216	0.183	0.364	0.206	0.000	0.000	0.000	**<0.001**
Cough	1459	0.187	0.172	0.330	0.287	0.888	0.000	0.000	**<0.001**
Erythema	1359	0.405	0.105	0.368	0.118	0.444	0.000	13.291	**<0.001**
Urticaria	949	0.249	0.090	0.157	0.106	0.000	44.307	0.000	**<0.001**
Hypersensitivity	893	0.109	0.108	0.204	0.137	1.332	0.000	0.000	**<0.001**
Anaphylactic shock	787	0.070	0.098	0.164	0.287	0.888	0.000	0.000	**<0.001**
Rash	769	0.146	0.080	0.170	0.131	1.332	0.000	0.000	**<0.001**
Blister	276	0.053	0.030	0.055	0.037	0.000	0.000	0.000	**0.001**
Erythema multiforme	261	0.048	0.031	0.036	0.025	0.444	0.000	0.000	**0.002**
Edema peripheral	243	0.031	0.025	0.086	0.069	0.000	0.000	0.000	**<0.001**
Face edema	234	0.031	0.029	0.048	0.019	0.444	0.000	0.000	**0.003**
Rash pruritic	192	0.045	0.019	0.034	0.044	0.000	0.000	0.000	**<0.001**
Peripheral swelling	159	0.024	0.016	0.052	0.044	0.000	0.000	0.000	**<0.001**
Asthmatic crisis	146	0.017	0.017	0.039	0.025	0.000	0.000	0.000	**0.032**
Bronchospasm	138	0.016	0.019	0.020	0.006	0.000	0.000	0.000	0.937
Dermatitis bullous	127	0.010	0.018	0.016	0.006	0.000	0.000	0.000	0.438
Laryngeal edema	114	0.014	0.014	0.025	0.006	0.000	0.000	0.000	0.541
Flushing	113	0.011	0.015	0.029	0.000	0.000	0.000	0.000	0.079
Lip edema	106	0.019	0.012	0.018	0.019	0.000	0.000	0.000	0.572
Edema	102	0.025	0.009	0.021	0.025	0.000	0.000	0.000	**<0.001**
Lip swelling	101	0.011	0.012	0.034	0.006	0.000	0.000	0.000	**0.002**
Eyelid edema	101	0.013	0.013	0.020	0.000	0.000	0.000	0.000	0.677

## Data Availability

Data available upon request.

## Supplementary Materials

The following supporting information can be downloaded at: https://www.mdpi.com/article/10.3390/life14060715/s1, Table S1: Method of grouping similar codes into main categories. The first line shows the category with respective codes below it; Table S2: All reaction codes presented with a descending number of cases. Incidence per 100,000 administered vaccine doses of allergy and hypersensitivity reactions to vaccines against COVID-19. Statistically significant differences (*p* < 0.05) are bolded.
