# Supplementary material for: Analysis of Allergy and Hypersensitivity Reactions to COVID-19 Vaccines According to the EudraVigilance Database
Source: Life (Basel). 2024 May 31;14(6):715. doi: 10.3390/life14060715 (PMC11205009; doi:10.3390/life14060715)
Supplement: Supplementary file 1 [file life-14-00715-s001.zip › life-3000581-supplementary.pdf]

Table S1. Method of grouping similar codes into main categories. The first line shows category with respective codes below it.

**Bronchial symptoms**	**Anaphylaxis**	**Rhinitis**	**Urticaria**	**Edema**	
Allergic cough	Anaphylactic reaction	Catarrh	Urticaria	Allergic oedema	Oedema genital
Cough	Anaphylactic shock	Rhinorrhoea		Angioedema	Oedema mouth
Asthmatic crisis	Anaphylactoid reaction	Sneezing		Blister	Oedema mucosal
Bronchial disorder	Anaphylactoid shock			Circumoral oedema	Oedema peripheral
Bronchial hyperreactivity	Shock			Ear swelling	Oral mucosal blistering
Bronchial obstruction	Shock symptom			Eye oedema	Oropharyngeal swelling
Bronchospasm				Eye swelling	Palatal oedema
Wheezing				Eyelid oedema	Palatal swelling
				Face oedema	Periorbital oedema
				Generalised oedema	Peripheral swelling
				Genital swelling	Pharyngeal oedema
				Injection site swelling	Respiratory tract oedema
				Laryngeal oedema	Scrotal oedema
				Lip oedema	Scrotal swelling
				Lip swelling	Swelling
				Localised oedema	Swelling face
				Mouth swelling	Swollen tongue
				Nasal oedema	Tongue oedema

Table S2. All reaction codes presented with descending number of cases. Incidence per 100,000 administered vaccine doses of allergy and hypersensitivity reactions to vaccines against COVID-19. Statistically significant differences ($p<0.05$) are bolded.

**All categories**									
Anaphylactic reaction	2018	0,176	0,258	0,454	0,374	1,775	44,307	0,000	**<0.001**
Angioedema	1564	0,216	0,183	0,364	0,206	0,000	0,000	0,000	**<0.001**
Cough	1459	0,187	0,172	0,330	0,287	0,888	0,000	0,000	**<0.001**
Erythema	1359	0,405	0,105	0,368	0,118	0,444	0,000	13,291	**<0.001**
Urticaria	949	0,249	0,090	0,157	0,106	0,000	44,307	0,000	**<0.001**
Hypersensitivity	893	0,109	0,108	0,204	0,137	1,332	0,000	0,000	**<0.001**
Anaphylactic shock	787	0,070	0,098	0,164	0,287	0,888	0,000	0,000	**<0.001**
Rash	769	0,146	0,080	0,170	0,131	1,332	0,000	0,000	**<0.001**
Blister	276	0,053	0,030	0,055	0,037	0,000	0,000	0,000	**0,001**
Erythema multiforme	261	0,048	0,031	0,036	0,025	0,444	0,000	0,000	**0,002**
Oedema peripheral	243	0,031	0,025	0,086	0,069	0,000	0,000	0,000	**<0.001**
Face oedema	234	0,031	0,029	0,048	0,019	0,444	0,000	0,000	**0,003**
Rash pruritic	192	0,045	0,019	0,034	0,044	0,000	0,000	0,000	**<0.001**
Peripheral swelling	159	0,024	0,016	0,052	0,044	0,000	0,000	0,000	**<0.001**
Asthmatic crisis	146	0,017	0,017	0,039	0,025	0,000	0,000	0,000	**0,032**
Bronchospasm	138	0,016	0,019	0,020	0,006	0,000	0,000	0,000	0,937
Dermatitis bullous	127	0,010	0,018	0,016	0,006	0,000	0,000	0,000	0,438
Laryngeal oedema	114	0,014	0,014	0,025	0,006	0,000	0,000	0,000	0,541
Flushing	113	0,011	0,015	0,029	0,000	0,000	0,000	0,000	0,079
Lip oedema	106	0,019	0,012	0,018	0,019	0,000	0,000	0,000	0,572
Oedema	102	0,025	0,009	0,021	0,025	0,000	0,000	0,000	**<0.001**
Lip swelling	101	0,011	0,012	0,034	0,006	0,000	0,000	0,000	**0,002**
Eyelid oedema	101	0,013	0,013	0,020	0,000	0,000	0,000	0,000	0,677
Hot flush	87	0,014	0,011	0,013	0,000	0,000	0,000	0,000	0,859
Rash maculo-papular	82	0,011	0,009	0,021	0,019	0,000	0,000	0,000	0,214
Swelling	78	0,029	0,006	0,007	0,000	0,000	0,000	0,000	**<0.001**
Anaphylactoid reaction	75	0,007	0,010	0,016	0,006	0,000	0,000	0,000	0,711
Eye swelling	72	0,009	0,008	0,023	0,006	0,000	0,000	0,000	**0,048**
Localised oedema	56	0,014	0,006	0,007	0,000	0,000	0,000	0,000	0,064
Rash erythematous	55	0,010	0,006	0,014	0,000	0,000	0,000	0,000	0,274
Swelling face	52	0,006	0,007	0,009	0,000	0,000	0,000	0,000	0,952
Drug eruption	48	0,005	0,006	0,013	0,000	0,000	0,000	0,000	0,520
Rash macular	42	0,006	0,004	0,013	0,019	0,000	0,000	0,000	0,064
Drug hypersensitivity	41	0,008	0,004	0,011	0,000	0,000	0,000	0,000	0,285
Acute generalised exanthematous pustulosis	36	0,003	0,005	0,005	0,013	0,000	0,000	0,000	0,816
Injection site swelling	35	0,005	0,004	0,014	0,000	0,000	0,000	0,000	0,038
Palatal oedema	35	0,006	0,004	0,002	0,013	0,000	0,000	0,000	0,686
Generalised oedema	32	0,006	0,003	0,009	0,000	0,444	0,000	0,000	**<0.001**
Type I hypersensitivity	32	0,005	0,004	0,000	0,000	0,000	0,000	0,000	0,744
Eye oedema	31	0,007	0,003	0,004	0,013	0,000	0,000	0,000	0,378
Dermatitis exfoliative generalised	30	0,006	0,003	0,005	0,000	0,000	0,000	0,000	0,817
Catarrh	28	0,002	0,004	0,005	0,006	0,000	0,000	0,000	0,867
Rash papular	28	0,005	0,003	0,004	0,006	0,000	0,000	0,000	0,951

Hypersensitivity vasculitis	25	0,003	0,003	0,007	0,000	0,000	0,000	0,000	0,772
Tongue oedema	22	0,002	0,002	0,013	0,000	0,000	0,000	0,000	**0,003**
Swollen tongue	21	0,002	0,003	0,005	0,000	0,000	0,000	0,000	0,921
Pharyngeal oedema	21	0,003	0,003	0,004	0,000	0,000	0,000	0,000	0,996
Rash vesicular	20	0,004	0,002	0,004	0,000	0,000	0,000	0,000	0,957
Bronchial hyperreactivity	20	0,005	0,002	0,002	0,000	0,000	0,000	0,000	0,849
Ear swelling	19	0,003	0,002	0,005	0,000	0,000	0,000	0,000	0,840
Laryngospasm	18	0,002	0,003	0,002	0,000	0,000	0,000	0,000	0,983
Toxic skin eruption	18	0,002	0,003	0,000	0,000	0,000	0,000	0,000	0,851
Allergic oedema	17	0,002	0,003	0,002	0,000	0,000	0,000	0,000	0,990
Drug reaction with eosinophilia and systemic symptoms	17	0,002	0,003	0,002	0,000	0,000	0,000	0,000	0,990
Stevens-Johnson syndrome	16	0,002	0,002	0,007	0,000	0,000	0,000	0,000	0,242
Injection site rash	15	0,006	0,000	0,007	0,006	0,000	0,000	0,000	**<0.001**
Allergic reaction to excipient	13	0,001	0,002	0,000	0,000	0,000	0,000	0,000	0,865
Mouth swelling	12	0,002	0,001	0,002	0,006	0,000	0,000	0,000	0,799
Shock	10	0,001	0,001	0,007	0,000	0,000	0,000	0,000	**0,014**
Bronchial obstruction	10	0,002	0,001	0,005	0,000	0,000	0,000	0,000	0,228
Photosensitivity reaction	10	0,002	0,001	0,005	0,006	0,000	0,000	0,000	0,033
Oedema mouth	10	0,001	0,001	0,002	0,006	0,000	0,000	0,000	0,752
Bronchial disorder	9	0,001	0,001	0,004	0,000	0,000	0,000	0,000	0,781
Rash morbilliform	9	0,002	0,001	0,004	0,000	0,000	0,000	0,000	0,745
Conjunctivitis allergic	8	0,000	0,001	0,004	0,000	0,000	0,000	0,000	0,541
Acute cutaneous lupus erythematosus	8	0,002	0,001	0,002	0,000	0,000	0,000	0,000	0,990
Allergic cough	7	0,002	0,001	0,004	0,000	0,000	0,000	0,000	0,428
Circumoral oedema	7	0,000	0,001	0,004	0,000	0,000	0,000	0,000	0,453
Periorbital oedema	7	0,002	0,001	0,002	0,000	0,000	0,000	0,000	0,966
Respiratory tract oedema	7	0,000	0,001	0,000	0,000	0,000	0,000	0,000	0,865
Toxic epidermal necrolysis	6	0,000	0,001	0,000	0,000	0,000	0,000	0,000	0,904
Type IV hypersensitivity reaction	6	0,004	0,000	0,000	0,000	0,000	0,000	0,000	0,005
Epiglottic oedema	5	0,002	0,000	0,002	0,000	0,000	0,000	0,000	0,744
Exfoliative rash	5	0,000	0,001	0,002	0,006	0,000	0,000	0,000	0,132
Symmetrical drug-related intertriginous and flexural exanthema	5	0,000	0,001	0,002	0,000	0,000	0,000	0,000	0,909
Vulval ulceration	5	0,000	0,001	0,002	0,000	0,000	0,000	0,000	0,909
Fixed eruption	5	0,001	0,001	0,000	0,000	0,000	0,000	0,000	0,998
Rhinorrhoea	5	0,001	0,001	0,000	0,006	0,000	0,000	0,000	0,222
Oral mucosal blistering	4	0,002	0,000	0,002	0,000	0,000	0,000	0,000	0,469
Genital ulceration	4	0,002	0,000	0,000	0,006	0,000	0,000	0,000	**0,028**
Skin exfoliation	4	0,000	0,001	0,000	0,000	0,000	0,000	0,000	0,963
Wheezing	3	0,000	0,000	0,004	0,000	0,000	0,000	0,000	**0,015**
Anaphylactoid shock	3	0,000	0,000	0,002	0,000	0,000	0,000	0,000	0,756
Sneezing	3	0,001	0,000	0,002	0,000	0,000	0,000	0,000	0,678

Dermatitis exfoliative	3	0,000	0,001	0,000	0,000	0,000	0,000	0,000	0,982
Oropharyngeal swelling	3	0,000	0,001	0,000	0,000	0,000	0,000	0,000	0,982
Tongue ulceration	3	0,000	0,001	0,000	0,000	0,000	0,000	0,000	0,982
Vasculitic rash	3	0,000	0,001	0,000	0,000	0,000	0,000	0,000	0,982
Scrotal oedema	2	0,001	0,000	0,002	0,000	0,000	0,000	0,000	0,253
Blister rupture	2	0,000	0,000	0,000	0,000	0,000	0,000	0,000	0,994
Eosinophil count increased	2	0,000	0,000	0,000	0,000	0,000	0,000	0,000	0,994
Genital swelling	2	0,001	0,000	0,000	0,000	0,000	0,000	0,000	0,953
Oedema mucosal	2	0,000	0,000	0,000	0,000	0,000	0,000	0,000	0,994
Type III immune complex mediated reaction	2	0,000	0,000	0,000	0,000	0,000	0,000	0,000	0,994
Upper airway obstruction	2	0,000	0,000	0,000	0,000	0,000	0,000	0,000	0,994
Mucosal ulceration	1	0,000	0,000	0,002	0,000	0,000	0,000	0,000	**0,047**
Nasal oedema	1	0,000	0,000	0,002	0,000	0,000	0,000	0,000	**0,047**
Oral mucosa erosion	1	0,000	0,000	0,002	0,000	0,000	0,000	0,000	**0,047**
Shock symptom	1	0,000	0,000	0,002	0,000	0,000	0,000	0,000	**0,047**
Stomatitis necrotising	1	0,000	0,000	0,002	0,000	0,000	0,000	0,000	**0,047**
Allergy test positive	1	0,001	0,000	0,000	0,000	0,000	0,000	0,000	0,567
Bronchial oedema	1	0,000	0,000	0,000	0,000	0,000	0,000	0,000	0,999
Eczema vesicular	1	0,000	0,000	0,000	0,000	0,000	0,000	0,000	0,999
Epidermal necrosis	1	0,000	0,000	0,000	0,000	0,000	0,000	0,000	0,999
Mucocutaneous rash	1	0,000	0,000	0,000	0,000	0,000	0,000	0,000	0,999
Oedema genital	1	0,000	0,000	0,000	0,000	0,000	0,000	0,000	0,999
Palatal swelling	1	0,001	0,000	0,000	0,000	0,000	0,000	0,000	0,567
Rash papulosquamous	1	0,000	0,000	0,000	0,000	0,000	0,000	0,000	0,999
Scrotal swelling	1	0,000	0,000	0,000	0,000	0,000	0,000	0,000	0,999
Skin erosion	1	0,000	0,000	0,000	0,000	0,000	0,000	0,000	0,999
Skin oedema	1	0,000	0,000	0,000	0,000	0,000	0,000	0,000	0,999
Upper respiratory tract congestion	1	0,000	0,000	0,000	0,000	0,000	0,000	0,000	0,999
Urticaria papular	1	0,001	0,000	0,000	0,000	0,000	0,000	0,000	0,567
